# 515. Optimizing Timing of Autologous Skin Cell Suspension After Enzymatic Debridement of Porcine Burns

**DOI:** 10.1093/jbcr/irag033.174

**Published:** 2026-04-06

**Authors:** Adam J Singer, Rachel B Bonn, Shi Fu, Huiting Luo, Zachery B Harris, Erica Heller, Steve McClain, Robert Ruzic, Eugene Jiang, Briman Yang, Miriam Rafailovich, Gurtej Singh, Marcia Simon, Steven Sandoval, Arash Karimi, M Hassan Arbab

**Affiliations:** Stony Brook University, Smithtown, NY; SUNY Stony Brook, Stony Brook, NY; SUNY Stony Brook, Stony Brook, NY; SUNY Stony Brook, Stony Brook, NY; SUNY Stony Brook, Stony Brook, NY; SUNY Stony Brook, Stony Brook, NY; SUNY Stony Brook, Stony Brook, NY; SUNY Stony Brook, Stony Brook, NY; SUNY Stony Brook, Stony Brook, NY; SUNY Stony Brook, Stony Brook, NY; SUNY Stony Brook, Stony Brook, NY; SUNY Stony Brook, Stony Brook, NY; SUNY Stony Brook, Stony Brook, NY; SUNY Stony Brook, Stony Brook, NY; SUNY Stony Brook, Stony Brook, NY; SUNY Stony Brook, Stony Brook, NY

## Abstract

**Introduction:**

Selective enzymatic burn debridement with a bromelain enriched agent derived from pineapple stems (anacaulase-bcdb) preserves viable dermis and may enable same-session autologous skin cell suspension (ASCS) application. We evaluated ASCS effectiveness after enzymatic debridement, the impact of application timing, and a noninvasive biomechanical device to monitor wound healing in a porcine burn model.

**Methods:**

We created 54 deep partial thickness burns on the backs of 4 anesthetized pigs. The wounds were enzymatically debrided at twenty-four hours and randomized to ASCS applied immediately, ASCS applied twenty-four hours later, or topical bacitracin. Wounds were photographed, and tissue biopsies were periodically obtained. Digital Image Speckle Correlation (DISC) was used to measure force propagation of a mechanical stimulus within the wounds. In vitro exposure assays tested the effect of residual enzymatic agent on human mesenchymal stromal cell viability across a graded concentration range.

**Results:**

ASCS improved percentage reepithelialization at day twenty-one (100% for both immediate and delayed ASCS vs. 87% for bacitracin, *p*=.047) and enhanced DISC-derived restoration of near-native wound biomechanics relative to topical antibiotics, supporting the clinical effectiveness of ASCS after enzymatic debridement. Comparisons between immediate and twenty-four-hour ASCS were inconclusive; group means were similar, but interpretation was limited by inter-animal variability in viable cell yield and unequal dose per area. In vitro, moderate to high but not low residual enzyme concentrations reduced cell viability, highlighting the need for thorough post-debridement rinsing to mitigate proteolytic carryover before cell application.

**Conclusions:**

Autologous skin cell suspension following bromelain-based enzymatic debridement of DPT porcine burns accelerates epithelial repair and functional biomechanical recovery in this model. Timing effects require confirmation under dose-normalized conditions with prospective control of cell yield and wound bed readiness.

**Applicability of Research to Practice:**

Our results suggest that ASCS can be applied to the enzymatically debrided wound bed of DPT burns, immediately after debridement as long as thorough cleansing of the wound bed significantly reduces proteolytic concentrations of the bromelain-based debridement agent. Application of ASCS appears to speed reepithelialization and partially restore the biomechanical characteristics of the wounds.

**Funding for the study:**

Supported by the Suffolk County Volunteer Firefighters Burn Fund, The Mathers Foundation and the SUNY Technology Accelerator Fund. The ASCS was donated by Avita Medical.

